# Case Report: Early-onset VEXAS syndrome with recurrent pulmonary inflammation and myelodysplasia: a diagnostic and therapeutic challenge

**DOI:** 10.3389/fimmu.2026.1737665

**Published:** 2026-02-03

**Authors:** Xianghong Jin, Xianyong Jiang, Yaping Liu, Miao Chen, Jiayuan Dai, Jin Xu, Min Shen

**Affiliations:** 1 Department of Rare Diseases, Peking Union Medical College Hospital, Chinese Academy of Medical Sciences & Peking Union Medical College, Beijing, China; 2 Department of Hematology, Peking Union Medical College Hospital, Chinese Academy of Medical Sciences & Peking Union Medical College, Beijing, China; 3 Center for Rare Diseases, Peking Union Medical College Hospital, Chinese Academy of Medical Sciences & Peking Union Medical College, Beijing, China

**Keywords:** clonal hematopoiesis, hematological-inflammatory diseases, pulmonary inflammation, UBA1 mutation, VEXAS syndrome

## Abstract

**Background:**

VEXAS (vacuoles, E1 enzyme, X-linked, autoinflammatory, somatic) syndrome is a newly recognized adult-onset autoinflammatory disorder caused by somatic mutations in the UBA1 gene. It typically presents in older males with systemic inflammation, hematologic abnormalities, and a relapsing, treatment-refractory course. Reports in younger patients remain rare, and pulmonary involvement is often misdiagnosed as infection.

**Case presentation:**

We present a 30-year-old man with a two-year history of recurrent scleritis, fever, cough, auricular chondritis, rash, and transfusion-dependent macrocytic anemia. Despite multiple immunosuppressants and broad-spectrum antimicrobials, symptoms persisted. Imaging revealed relapsing bilateral ground-glass pulmonary infiltrates, and repeated microbiologic studies were negative. Bone marrow showed vacuolated precursors and cytogenetic abnormalities. A somatic UBA1 mutation (NM_003334 exon3 c.121A>G p.M41V) was detected with high variant allele frequency in blood and marrow, and notably, also in bronchoalveolar lavage cells. The patient was diagnosed with VEXAS syndrome with early myelodysplastic features. Tocilizumab induced transient improvement, but disease relapse followed. He is currently being evaluated for allogeneic hematopoietic stem cell transplantation (AHSCT).

**Conclusion:**

This case represents one of the youngest patients reported with VEXAS syndrome and provides rare evidence of UBA1 mutation in pulmonary cells, supporting the concept of tissue-level clonal inflammation. It highlights the importance of considering VEXAS in younger patients with unexplained systemic inflammation, cytopenias, and non-infectious pulmonary infiltrates, and supports early genetic testing and multidisciplinary management.

## Case description

**Figure 1:**
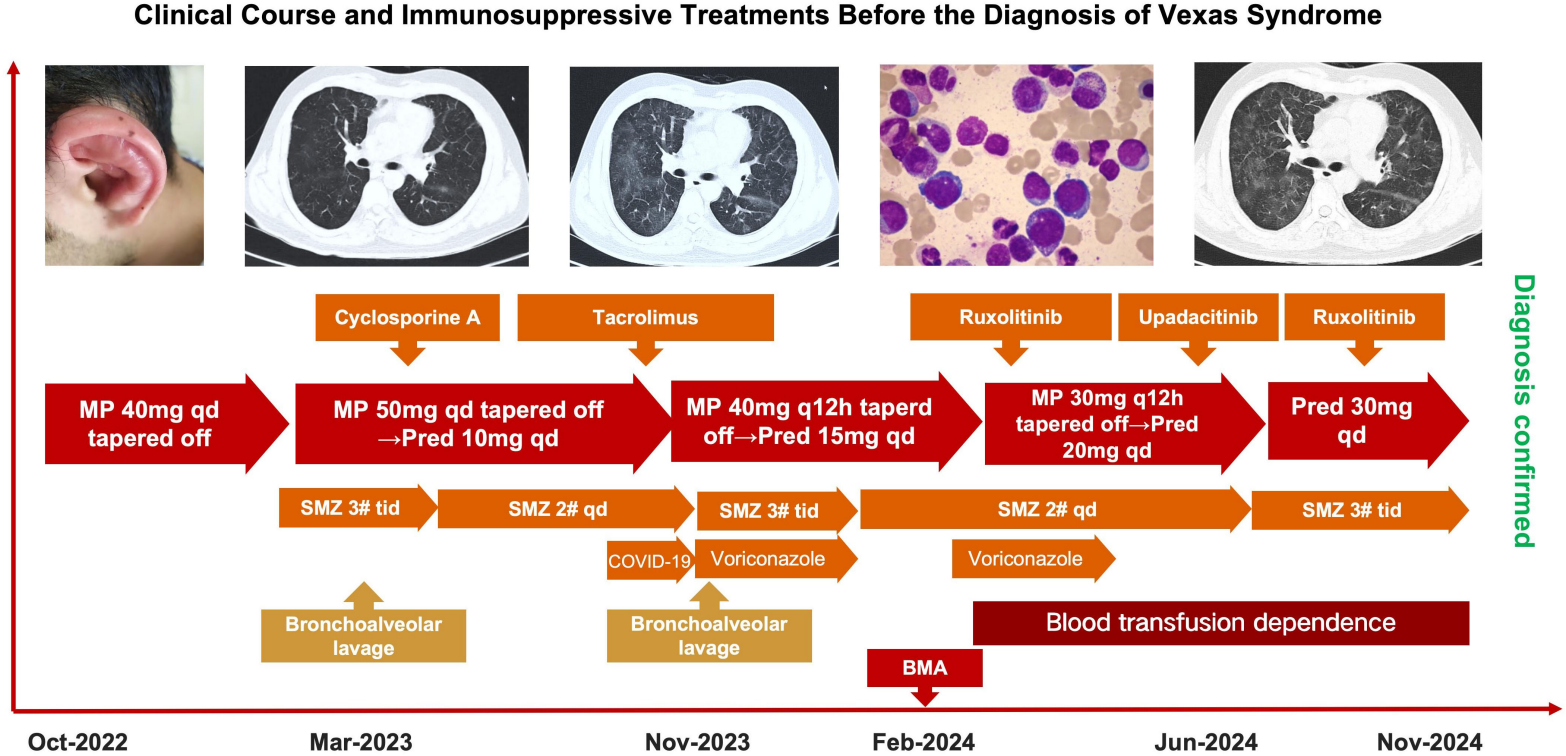
Clinical course and immunosuppressive treatments prior to diagnosis of VEXAS syndrome. Timeline showing disease progression and immunosuppressive therapies prior to diagnosis of VEXAS. Despite sequential use of corticosteroids and multiple immunomodulators, symptoms persisted. CT scans show recurrent pulmonary infiltrates. Bone marrow aspiration (BMA) revealed vacuolated precursors. Diagnosis was confirmed following detection of *UBA1* mutation in peripheral blood and BALF. MP, methylprednisolone; Pred, prednisone; SMZ, sulfamethoxazole; BAL, bronchoalveolar lavage; BMA, bone marrow aspiration; CT, computed tomography; Rux, ruxolitinib; Upa, upadacitinib; CsA, cyclosporine A; Tac, tacrolimus.

A 30-year-old Han Chinese man, the only child of healthy, non-consanguineous parents, was admitted in September 2024 with a two-year history of recurrent scleritis, productive cough, and moderate-grade fever, along with a six-month history of worsening fatigue. He also presented with bilateral auricular swelling and erythema, nasal bridge redness, non-erosive arthritis, and a transient papular rash. Early in the disease course, he developed a diffuse pruritic erythematous papular eruption involving the trunk and extremities. A skin biopsy at that time showed scattered perivascular and periadnexal lymphocytic infiltration in the superficial dermis. The rash resolved with intravenous corticosteroids and did not recur. While no photographs were taken during the acute flare, an image from the convalescent phase is provided (**Supplementary Figure 1**). The patient also experienced recurrent conjunctival hyperemia and periorbital swelling, for which he was evaluated by an ophthalmologist and diagnosed with anterior uveitis. Initial corticosteroid therapy (methylprednisolone 40–80 mg/day) led to transient remission, but symptoms relapsed during tapering (**Figure 1**). Besides, he had no significant past medical history and no family history of autoimmune, hematologic, or genetic disorders. There was no known consanguinity or evidence of primary immunodeficiency in childhood. He had completed all routine childhood vaccinations in accordance with the national immunization schedule without any adverse reactions. He reported no relevant environmental exposures or alcohol use and had not received immunosuppressive treatment prior to disease onset.

Initial evaluation raised suspicion for pneumonia, but repeated courses of broad-spectrum antimicrobials, including piperacillin-tazobactam and meropenem, yielded no clinical improvement. Extensive infectious workups were performed multiple times throughout the disease course. These included aerobic and anaerobic blood and sputum cultures, fungal cultures, and molecular assays for Mycobacterium tuberculosis (PCR and culture). Serum galactomannan and (1–3)-β-D-glucan tests were negative. Multiplex PCR panels for viral pathogens—including cytomegalovirus (CMV), Epstein–Barr virus (EBV), herpes simplex virus (HSV), human herpesvirus 6 (HHV-6), adenovirus, and respiratory viruses—were also negative. No infectious etiology was identified during several hospitalizations. Laboratory data demonstrated progressive macrocytic anemia (hemoglobin declining from 110 g/L to 40 g/L), elevated inflammatory markers (CRP 146 mg/L [reference <8], ESR >100 mm/h [reference: 0–15], ferritin 1031 ng/mL [reference: 24–336], IL-6 >100 pg/mL [reference <5.9]), and transfusion dependence. Autoimmune and immunologic evaluations revealed negative antinuclear antibody (ANA, titer <1:80), negative anti-neutrophil cytoplasmic antibodies (ANCA), and normal complement levels (C3: 1.44 g/L [reference: 0.73–1.66], C4: 0.287 g/L [reference: 0.1–0.4]). Serum immunoglobulins were within normal ranges (IgG: 8.31 g/L [reference: 7–17], IgA: 1.58 g/L [reference: 0.7–4], IgM: 1.11 g/L [reference: 0.4–2.3]). Lymphocyte immunophenotyping revealed CD3+ T cells at 2073/μL (reference: 1185–1901), CD4+ T cells 1127/μL (561–1137), CD8+ T cells 851/μL (404–754), CD19+ B cells 397/μL (180–324), and CD56+ NK cells 33/μL (175–567).

Chest CT demonstrated recurrent bilateral ground-glass opacities (**Figure 1**). Pulmonary function testing revealed restrictive changes and reduced diffusion capacity. Bronchoalveolar lavage fluid (BALF) consistently tested negative for bacteria, fungi, mycobacteria, and viruses. Importantly, genetic analysis began with whole-exome sequencing (WES) of peripheral blood at the time of suspected VEXAS syndrome, which revealed a somatic mutation in UBA1 (NM_003334, exon 3, c.121A>G; p.Met41Val). Laboratory data demonstrated progressively worsening anemia. The earliest available complete blood count, dated March 10, 2023, showed normocytic normochromic anemia with hemoglobin of 110 g/L. Macrocytic indices were first documented on February 14, 2024 (HGB 85 g/L, MCV 106.4 fL, MCH 36 pg, MCHC 339 g/L). Common causes of macrocytosis were systematically excluded: serum vitamin B12 and folate levels were within normal ranges; liver and thyroid function tests were unremarkable; hemolysis markers (LDH, haptoglobin, Coombs tests) were negative; and the patient reported no alcohol consumption. These findings effectively ruled out infectious, autoimmune, and primary immunodeficiency causes. By March 2024, he developed transfusion-dependent anemia. Bone marrow aspiration revealed hypercellularity with vacuolization in myeloid and erythroid precursors (**Figure 1**) and dysplasia in both erythroid and granulocytic lineages and 2.5% blasts. Molecular testing confirmed a somatic UBA1 mutation with variant allele frequency of 92% in marrow. Bone marrow karyotype was otherwise normal. Targeted next-generation sequencing (NGS) of marrow DNA detected no mutations in IPSS-M–associated genes. Based on recurrent systemic inflammation, pulmonary infiltrates, vacuolization, and UBA1 mutation, a diagnosis of VEXAS syndrome was established, with concurrent myelodysplastic changes. Although not meeting classical diagnostic criteria for primary myelodysplastic syndrome (MDS), these findings were consistent with VEXAS-associated marrow dysplasia with multilineage involvement. Besides, targeted next-generation sequencing (NGS) was performed on bone marrow and bronchoalveolar lavage fluid (BALF) using a myeloid panel that included IPSS-M–related genes. The UBA1 mutation was consistently detected across all specimens (peripheral blood, bone marrow, and BALF-derived cells), with no additional somatic variants identified.

**Figure 2:**
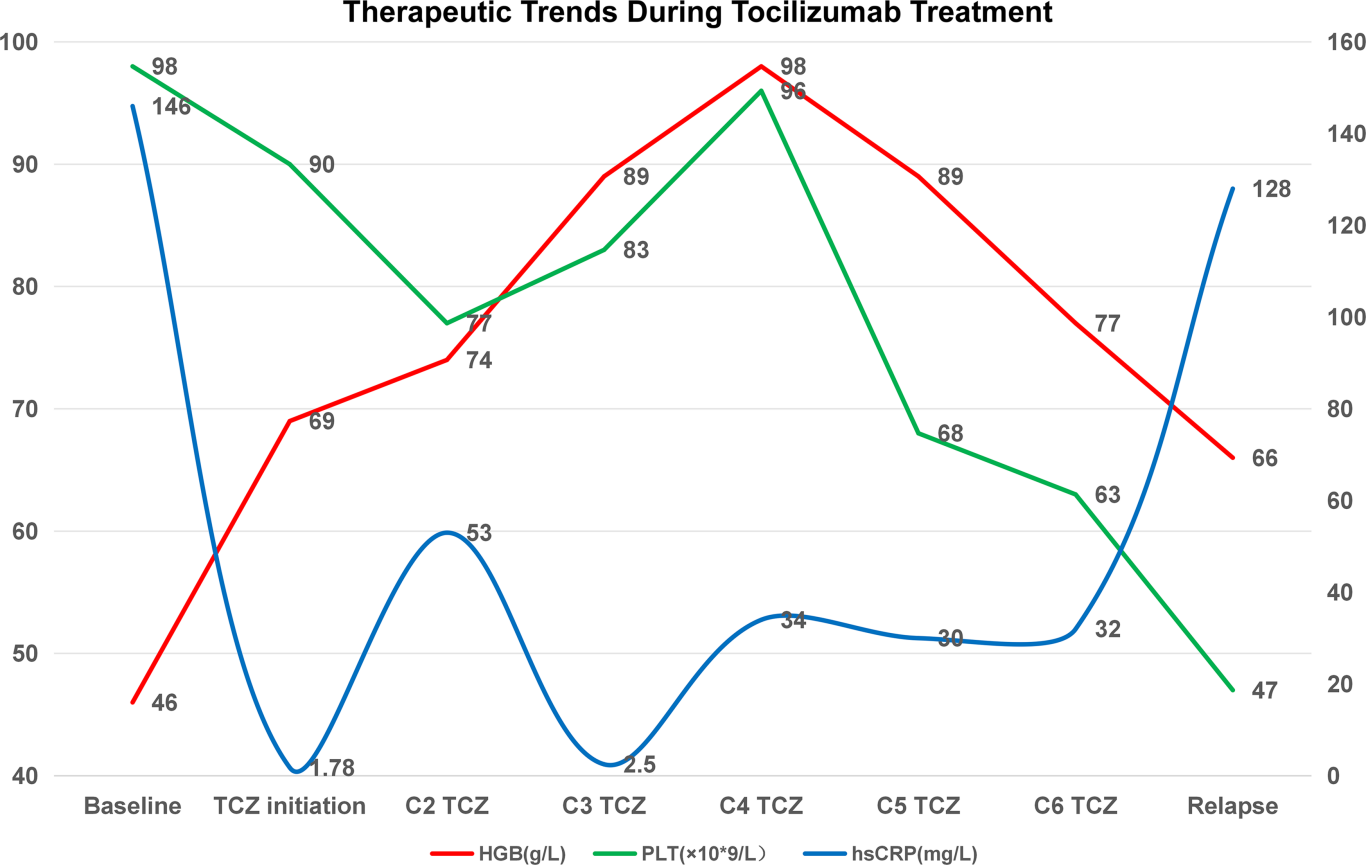
Laboratory trends during tocilizumab (TCZ) treatment. During six cycles of TCZ, hsCRP decreased significantly, and hemoglobin improved with temporary transfusion independence. Platelet count declined progressively. Inflammatory relapse occurred after treatment cessation, indicating partial and time-limited response. TCZ, tocilizumab; HGB, hemoglobin; PLT, platelet count; hsCRP, high-sensitivity C-reactive protein.

Treatment with tocilizumab (8 mg/kg every four weeks) led to rapid improvement in fever, cough, fatigue, inflammatory markers, and pulmonary opacities, and the patient achieved temporary transfusion independence (**Figure 2**). However, relapse occurred after six cycles, with recurrent anemia and inflammatory activity. Azacitidine therapy (75 mg/m² for 7 days every 28 days) was initiated on May 25, 2025, but has been interrupted due to episodes of pulmonary and urinary tract infections. As of submission, the patient has completed four cycles. While hemoglobin levels remain low (50–70 g/L). The patient remains transfusion dependent and is undergoing evaluation for allogeneic stem cell transplantation.

## Discussion

VEXAS syndrome, first described in 2020, is a late-onset autoinflammatory condition driven by somatic mutations in the *UBA1* gene on the X chromosome (1), often arising in hematopoietic stem cells (2). It typically presents in men over 60 years of age, with a constellation of systemic inflammatory features and hematological abnormalities, notably macrocytic anemia, cytopenia, and vacuolization of myeloid and erythroid precursors (2). The clinical course is often relapsing and treatment-refractory, with variable progression toward myelodysplastic syndromes (MDS) or other clonal hematologic conditions (3). As recognition of VEXAS increases, its phenotypic diversity is becoming more apparent, challenging assumptions about age, organ involvement, and treatment response.

Recent reports, including a 29-year-old patient with VEXAS and myelodysplastic syndrome (4), suggest that the age of onset may be younger than initially appreciated. Our case further supports this evolving understanding, with onset at just 30 years of age. The presence of relapsing polychondritis, pulmonary infiltrates, and cytopenias in a young male underscores the risk of diagnostic delay when clinical suspicion is limited by age bias.The identification of the UBA1 p.Met41Val mutation in BALF-derived cells suggests that UBA1-mutated myeloid clones may infiltrate the lung and contribute to local inflammatory responses. While this does not confirm clonal expansion within the pulmonary compartment, it supports the hypothesis that pulmonary involvement in VEXAS may result from the activation and cytokine-mediated effects of circulating mutant hematopoietic cells. Together, these findings emphasize that VEXAS should be considered in younger patients with unexplained systemic inflammation and marrow abnormalities, and that early genetic testing is essential for timely diagnosis and management.

The identification of the *UBA1* p.Met41Val mutation in BAL cells—alongside peripheral blood and bone marrow—supports a mechanistic link between clonal hematopoiesis and organ-specific inflammation. To our knowledge, this is among the first cases to confirm such a mutation in BAL-derived cells, suggesting that tissue-resident macrophages may contribute to the pulmonary manifestations of VEXAS. The persistently negative microbiologic testing and poor response to antimicrobials further support a non-infectious, systemic inflammatory etiology. While peripheral blood remains the primary sample for detecting UBA1 mutations, site-specific testing of BALF may aid in challenging cases with nonresolving pulmonary infiltrates and inconclusive infectious or autoimmune workup. In this case, identification of the same UBA1 mutation in BALF-derived cells supported clonal myeloid infiltration as a contributor to lung inflammation, helping to redirect management away from ineffective antimicrobial therapies. Given the invasiveness and technical limitations of BALF sampling, this approach is best reserved for select patients with diagnostic uncertainty, rather than as a routine alternative to blood-based testing.

Therapeutically, the patient exhibited refractoriness to multiple conventional immunosuppressants, including corticosteroids, calcineurin inhibitors, and JAK inhibitors, underscoring the difficulty in achieving durable disease control with standard agents. Tocilizumab, an IL-6 receptor antagonist, led to partial clinical and biochemical remission, including transfusion independence, but relapse occurred after six cycles, aligning with recent data suggesting IL-6 blockade might offer temporary benefit without altering clonal disease progression. Azacitidine, a hypomethylating agent used in MDS, produced minimal benefit. These findings reinforce the concept that while cytokine modulation may provide symptomatic relief (5–8), disease modification likely requires targeting the underlying clonal process.

Given the patient’s young age and early evolution toward MDS, AHSCT remains the most promising curative option and should be considered early in similar presentations (9–11). For VEXAS patients with clonal hematologic involvement, the indications and timing for AHSCT should be determined by an integrated assessment of clonal disease and systemic inflammation rather than conventional MDS risk stratification alone. Emerging data suggest that standard scores such as IPSS-R or IPSS-M may underestimate risk in UBA1-mutated MDS, because they do not account for the high inflammatory burden, cumulative toxicities of prolonged immunosuppression, and organ damage that characterize VEXAS, even in the absence of high-risk cytogenetic or molecular lesions (12). In this context, persistent transfusion-dependent cytopenias, progressive marrow failure, clonal evolution, or excess blasts remain classical triggers to consider transplantation, but in VEXAS they must be weighed together with steroid or JAK/IL-6 inhibitor dependence, recurrent or life-threatening infections, and organ-threatening manifestations such as refractory pulmonary infiltrates or vasculitis. For younger and otherwise fit patients, particularly those with early MDS or clearly defined clonal cytopenia, many experts now advocate for earlier referral to transplantation before irreversible organ damage or treatment-related frailty develops, accepting a transplant-related risk that is justified by the high long-term morbidity and mortality associated with uncontrolled clonal inflammation. Our case illustrates this principle, as a young patient with evolving MDS, transfusion dependence, and refractory systemic inflammation in the setting of UBA1 mutation, for whom early consideration of allogeneic transplantation is warranted despite ostensibly lower-risk MDS features.

This case also underscores the importance of multidisciplinary collaboration across rheumatology, hematology, pulmonology, and clinical genetics. The convergence of systemic inflammation, pulmonary disease, and hematologic changes demands coordinated diagnostic pathways and therapeutic strategies. As the understanding of VEXAS deepens, integrating molecular diagnostics into earlier stages of clinical evaluation may accelerate recognition and improve outcomes, particularly in atypical or treatment-resistant presentations.

## Conclusions

This report describes one of the youngest patients with VEXAS syndrome, with unique evidence of *UBA1* mutation in BAL cells, early myelodysplastic features, and refractoriness to multiple therapies. The case expands the clinical spectrum of VEXAS, provides mechanistic insights into pulmonary disease, and highlights the importance of genetic testing, multidisciplinary team (MDT) collaboration, and early consideration of transplantation.

## Data Availability

The original contributions presented in the study are included in the article/**Supplementary Material**. Further inquiries can be directed to the corresponding authors.

## Supplementary material

Supplementary Figure 1 Fading erythematous papular rash on the trunk during recovery, following resolution of acute inflammation after intravenous corticosteroid therapy. Skin biopsy during the active phase revealed superficial perivascular and periadnexal lymphocytic infiltration.
