# Supplementary figures and images for: Case Report: Early-onset VEXAS syndrome with recurrent pulmonary inflammation and myelodysplasia: a diagnostic and therapeutic challenge
Source: Front Immunol. 2026 Feb 3;17:1737665. doi: 10.3389/fimmu.2026.1737665 (PMC12910313; doi:10.3389/fimmu.2026.1737665)

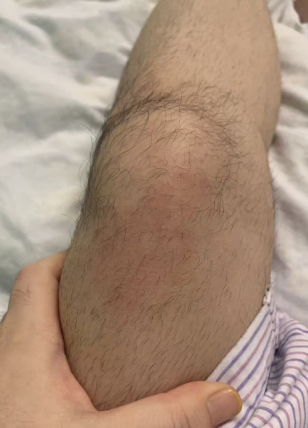

Supplement: Supplementary Figure 1 — Fading erythematous papular rash on the trunk during recovery, following resolution of acute inflammation after intravenous corticosteroid therapy. Skin biopsy during the active phase revealed superficial perivascular and periadnexal lymphocytic infiltration. [file Image1.png]
